# RETRACTED ARTICLE: Fetal methotrexate syndrome and Antley–Bixler syndrome should not be confused

**DOI:** 10.1007/s00247-018-4125-9

**Published:** 2018-04-19

**Authors:** C. Richards, Christine M. Hall, D. Johnson, Amaka C. Offiah

**Affiliations:** 1 University of Sheffield Medical School, Sheffield, UK; 2 Great Ormond Street Hospital for Children (retired), London, UK; 3 Sheffield Children’s NHS Foundation Trust, Sheffield, UK; 4 University of Sheffield, Academic Unit of Child Health, Sheffield Children’s NHS Foundation Trust, Western Bank, Damer Street Building, Sheffield, S10 2TH UK

The authors have retracted this article because the legal guardians of the patient described have withdrawn consent for publication of the case and images. The scientific content of the article has been removed to protect the patient’s privacy. All authors agree to this retraction.

